# Meat Consumption and Risk of Metabolic Syndrome: Results from the Korean Population and a Meta-Analysis of Observational Studies

**DOI:** 10.3390/nu10040390

**Published:** 2018-03-22

**Authors:** Youngyo Kim, Youjin Je

**Affiliations:** Department of Food and Nutrition, Kyung Hee University, Seoul 02447, Korea; youngyokim06@gmail.com

**Keywords:** metabolic syndrome, processed meat, white meat, meta-analysis, red meat

## Abstract

Many studies have reported harmful effects of red meat or processed meat on chronic diseases including cancer and diabetes, but epidemiological evidence for metabolic syndrome is limited and remains controversial. Therefore, we performed a meta-analysis of observational studies to assess the association between various meat consumption and risk of metabolic syndrome. The PubMed and ISI Web of Science databases were searched through June 2017, and further included unpublished results from Korea National Health and Nutrition Examination Survey 2012–2015, including 8387 Korean adults. Sixteen studies were suitable for meta-analysis, which included 19,579 cases among 76,111 participants. We used a random-effects model to calculate the pooled relative risks (RR) and 95% confidence intervals (CI). The pooled RR for metabolic syndrome of the highest versus lowest category of meat intake was 1.14 (95% CI: 1.05, 1.23) for total meat, 1.33 (95% CI: 1.01, 1.74) for red meat, 1.35 (95% CI: 1.18, 1.54) for processed meat, and 0.86 (95% CI: 0.76, 0.97) for white meat. All of these associations did not differ significantly by study design and adjustment factors. Our findings indicated that total, red, and processed meat intake is positively associated with metabolic syndrome, and white meat intake is inversely associated with metabolic syndrome.

## 1. Introduction

A significant increase in the prevalence of metabolic syndrome has been observed worldwide [1]. Metabolic syndrome consists of an aggregation of metabolic abnormalities including central obesity, hypertriglyceridemia, hyperglycemia, low HDL cholesterol levels, and high blood pressure. These metabolic disorders are also risk factors of type 2 diabetes and cardiovascular disease (CVD), and a large body of evidence suggested that metabolic syndrome is positively related to high risks of CVD [2], type 2 diabetes [3], specific cancers [4], and total mortality [5]. Due to its high prevalence, the development of a preventive strategy against metabolic syndrome is needed to improve public health. The identification of modifiable factors affecting metabolic syndrome may help decrease the burden of death from CVD, type 2 diabetes, and several cancers. 

Accumulating evidence from previous studies indicated that a high intake of red meat and processed meat could raise the risk of chronic diseases, including type 2 diabetes [6], CVD [7,8], and several types of cancer [9,10,11,12]. However, the associations between the consumption of red meat, processed meat and white meat and the risk of metabolic syndrome remain largely inconclusive. One cross-sectional study first provided a non-significant positive association between total meat intake and metabolic syndrome in data from an Epidemiology study on the Insulin Resistance Syndrome (DESIR) [13]. Several subsequent observational studies have investigated the relationship between red, processed, and white meat consumption and metabolic syndrome, but the results are sparse and inconsistent [14,15,16,17,18,19,20,21,22,23,24]. 

Therefore, we conducted a systematic review and comprehensive meta-analysis to quantitatively evaluate the relationship between meat intake and metabolic syndrome. In addition, we analyzed the association using the data of Korea National Health and Nutrition Examination Survey (KNHANES) and included these new results in our meta-analysis.

## 2. Materials and Methods 

### 2.1. Literature Search

We searched the electronic databases (PubMed and ISI Web of Science) through June 2017 to identify eligible studies published in English as full-length articles. The search terms were the following: “(meat OR beef OR pork OR veal OR lamb OR steak OR hamburger OR ham OR bacon OR sausage OR poultry OR chicken OR turkey) combined with (metabolic syndrome OR insulin resistant syndrome OR syndrome X).” The reference lists of retrieved articles or published reviews were also manually screened to search further relevant studies. ‘Red meat’ included red meat and processed red meat. ‘White meat’ included poultry. In the study conducted by Cocate et al. [14], fish was included in the white meat consumption group and thus we performed a sensitivity analysis by removing this study. ‘Processed meat’ included offal, ham, sausages, pate, hamburgers, bacon and sundae. ‘Total meat’ included the total of these three categories. If the study did not report information for the meat category, we included the results in the meta-analysis of total meat consumption.

### 2.2. Study Selection

Studies were included in the current meta-analysis when they met the following criteria: (1) they were epidemiological studies; (2) the exposure of interest was meat consumption; (3) the outcome of interest was defined as prevalence or incidence of metabolic syndrome; and (4) they provided relative risks (RR) and 95% confidence intervals (CI). Studies targeting people with disease were excluded. 

### 2.3. Data Extraction

Data were extracted according to the meta-analysis of observational studies in epidemiology (MOOSE) guidelines [25] by two investigators (Y.K. and Y.J.). The following information from all studies was extracted: first author name; year of publication; design of study; country; study period or follow-up period; number of events/participants or person-years; sex; covariates used for adjustment; RRs and 95% CIs for the relationship between meat intake and metabolic syndrome across all categories of exposure. If the studies reported various RRs on this association, the RR from the most fully adjustment was used. 

To include all available data in the meta-analysis, we investigated the association between meat consumption and metabolic syndrome in KNHANES 2012–2015 and conducted a meta-analysis including these results. The KNHANES is a cross-sectional, nationally representative sample of the South Korean population that uses a multistage probability sampling design to represent the non-institutionalized civilian South Korean population. The KNHANES assessed the health and nutritional status of Koreans via 3 surveys including a health examination, a health interview, and a nutrition survey. The procedures of human subjects for KNHANES were approved by the Institutional Review Board of Korea Centers for Disease Control and Prevention, and informed consent was received from all subjects. Dietary consumption was assessed using the 112-item dish-based semi-quantitative food frequency questionnaires (FFQ) of KNHANES. Details of the study populations and methods are provided in the online-only Data Supplement.

### 2.4. Statistical Analysis

In the present meta-analysis, odds ratios (ORs) and hazard ratios (HRs) were considered equivalent to RRs [26]. The natural logarithm values of the RRs from the original study were combined using the DerSimonian and Laird random-effects models, which take into account within- and between-study variations, to obtain the pooled estimate of metabolic syndrome for the highest vs. the lowest levels of meat intake [27]. We recalculated the RR and its 95% CI when a study did not report the lowest level as a reference [23]. We presented the summary estimates as forest plots where the extent of data markers (squares) is consistent with the inverse of the variance of the natural logarithm of RR from an individual study, and the diamond displays a pooled RR. Statistical heterogeneity between studies was assessed through the *Q* [28] and *I^2^* statistics [29]. The subgroup analysis was performed by design of study, geographical region, and differences in adjustment factors. To test whether the results were not simply attributable to the inclusion of a single study, we conducted sensitivity analyses by removing one study at a time. We evaluated publication bias through Egger’s test [30] and Begg’s test [31]. All statistical analyses were performed using Stata software (version 14.2; StataCorp (College Station, TX, USA)). A two-tailed *p*-value less than 0.05 was deemed statistically significant.

## 3. Results

### 3.1. KNHANES Analysis

The characteristics of study populations by meat consumption are present in Supplementary Table S1. Subjects in the highest quintile of meat intake were younger, drank more alcohol, and had a high education level than those in the lowest quintile. The association between white meat intake and metabolic syndrome is shown in Supplementary Table S2. In the overall population, subjects in the highest quintile of white meat consumption had a decreased prevalence of hypertriglyceridemia (OR = 0.77, 95% CI: 0.61, 0.99, P-trend = 0.010) and elevated blood pressure (OR = 0.67, 95% CI: 0.50, 0.89, P-trend= 0.005) compared to those in the lowest quintile after controlling for potential confounders. The association between red meat consumption and metabolic syndrome is shown in Supplementary Table S3. In a comparison of highest vs. lowest consumption, we found no significant association between red meat intake and the prevalence of metabolic syndrome and its components. The association between processed meat consumption and metabolic syndrome is shown in Supplementary Table S4. Compared to those in the first quintile, subjects in the fifth quintile of processed meat consumption had a higher prevalence of hyperglycemia (OR = 1.27, 95% CI: 1.00, 1.60).

### 3.2. Systematic Review and Meta-Analysis

#### 3.2.1. Study Characteristics

**Figure 1:**
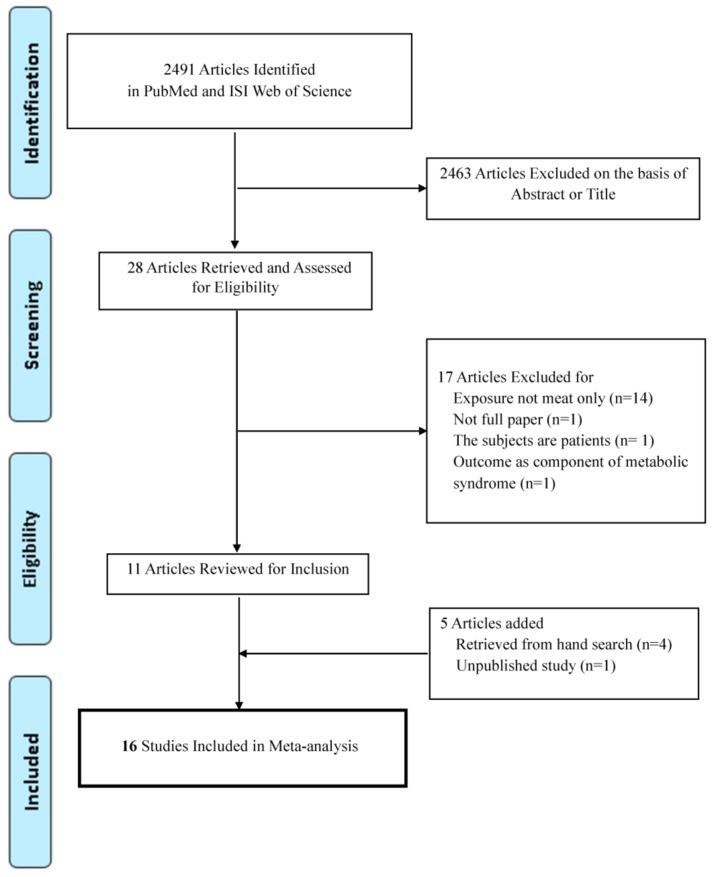
Flow chart of the selection of studies included in the meta-analysis.

Sixteen studies including 19,579 cases among 76,111 participants were suitable for meta-analysis (Figure 1) [13,14,15,16,17,18,19,21,22,23,24,32,33,34,35]. The characteristics of studies which were included in meta-analysis are presented in Table 1. All articles were published from 2000 to 2017. By geographic region, 7 studies were performed in Asia [15,23,32,33,34,35], 5 in Europe [13,16,17,19,24], 3 in North or South America [14,21,22], and 1 in the Middle East [18]. Most of the studies investigated food intake using FFQ, and some studies used a food record [17,24] or questionnaire [23,32,34]. The criteria used to define metabolic syndrome were mostly NCEP ATP III criteria or Harmonized criteria from the International Diabetes Federation and the American Heart Association/National Heart, Lung, and Blood Institute [3]. Most studies reported risk estimates adjusted for age [13,14,15,16,17,18,19,21,22,24,32,33,34,35], smoking [14,15,16,17,19,21,22,24,35], alcohol [13,14,15,16,17,18,19,22,24,35], and physical activity [13,14,15,16,18,19,21,22,24,35]. 

#### 3.2.2. Total Meat Consumption and Metabolic Syndrome

**Figure 2:**
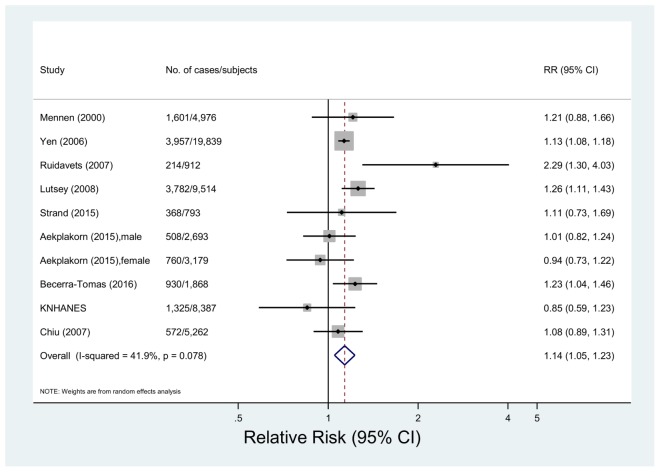
Forest plot of observational studies of metabolic syndrome for the highest vs. lowest levels of total meat consumption, using a random-effects model.

Nine studies including 52,733 subjects and 18,135 events of metabolic syndrome were eligible for the meta-analysis of total meat consumption and metabolic syndrome [13,15,19,21,23,24,32,34]. The multivariable-adjusted pooled RR was 1.14 (95% CI 1.05, 1.23) with no significant heterogeneity among studies (*p* = 0.08, *I*^2^ = 41.9%) (Figure 2). In a sensitivity analysis by removing one study at a time, the pooled RRs were between 1.11 (95% CI: 1.02, 1.21) and 1.15 (95% CI: 1.06, 1.25) (data not shown). The variances by study design, region, adjustment factors or meat intake assessment were insignificant (*p* > 0.10 for all comparisons) (Table 2). 

#### 3.2.3. Red Meat Consumption and Metabolic Syndrome

**Figure 3:**
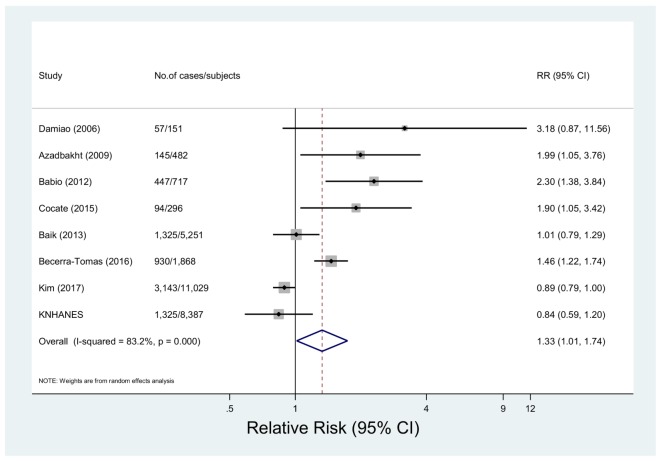
Forest plot of observational studies of metabolic syndrome for the highest vs. lowest levels of red meat consumption, using a random-effects model.

Eight studies with 7466 cases among 28,181 subjects investigated the relationship between red meat intake and metabolic syndrome [14,16,18,19,22,33,35]. The multivariable-adjusted pooled RR was 1.33 (95% CI 1.01, 1.74) showing a high degree of heterogeneity (*p* < 0.001, *I*^2^ = 83.2%) (Figure 3). In the sensitivity analysis, the summary RRs were between 1.22 (95% CI: 0.94, 1.60) and 1.44 (95% CI: 1.07, 1.95) (data not shown). By geographical region, a positive association was found for the studies carried out in Europe (RR = 1.72, 95% CI: 1.12, 2.63), South America (RR = 2.08, 95% CI: 1.22, 3.55), and the Middle East (RR = 1.99, 95% CI: 1.05, 3.76), while the inverse association was shown for studies conducted in Asia (RR = 0.91, 95% CI: 0.82, 1.00) (*p* for Europe, South America, or Middle East vs. Asia = 0.01, 0.04, and 0.08, respectively). By meat intake assessment, studies which assessed meat intake by grams showed a positive association (RR=1.71, 95% CI: 1.37, 2.12), while studies which assessed meat intake by servings or frequencies showed the inverse association (RR = 0.91, 95% CI: 0.82, 1.00) (*p* = 0.002). There was no significant difference by study design or adjustment factors (*p* > 0.33 for all comparisons) (Table 2).

#### 3.2.4. Processed Meat Consumption and Metabolic Syndrome

**Figure 4:**
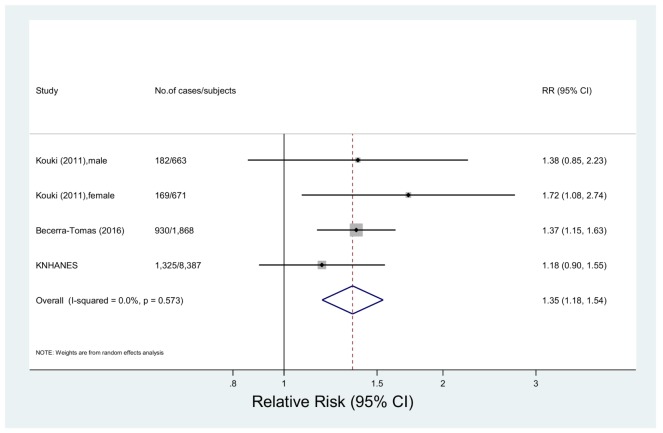
Forest plot of observational studies of metabolic syndrome for the highest vs. lowest levels of processed meat consumption, using a random-effects model.

Three studies including 11,589 subjects and 2606 cases were included in the meta-analysis of processed meat intake and metabolic syndrome [17,19]. The multivariable-adjusted pooled RR was 1.35 (95% CI: 1.18, 1.54) with no significant heterogeneity (*p* = 0.57, *I*^2^ = 0.0%) (Figure 4). The summary RRs were between 1.32 (95% CI: 1.06, 1.63) and 1.40 (95% CI: 1.21, 1.64) in the sensitivity analysis (data not shown). 

#### 3.2.5. White Meat Consumption and Metabolic Syndrome

**Figure 5:**
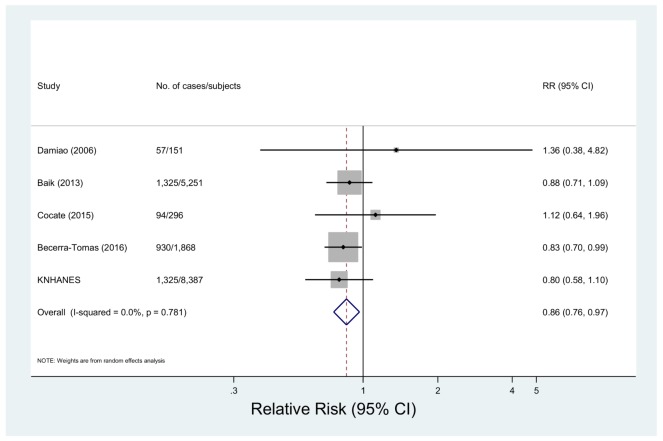
Forest plot of observational studies of metabolic syndrome for the highest vs. lowest levels of white meat consumption, using a random-effects model.

Five studies with 3731 cases among 15,953 subjects investigated the association between white meat intake and metabolic syndrome [14,19,22,35]. The multivariable-adjusted pooled RR was 0.86 (95% CI: 0.76, 0.97) with non-significant heterogeneity (*p* = 0.78, *I*^2^ = 0.0%) (Figure 5). In the sensitivity analysis, the summary RRs were between 0.85 (95% CI: 0.73, 0.98) and 0.88 (95% CI: 0.74, 1.04) (data not shown). The differences by study design, region, adjustment factors or meat intake assessment were insignificant (*p* > 0.3 for all comparisons) (Table 2). 

#### 3.2.6. Publication Bias

We found no evidence of publication bias for the meta-analysis of total meat (Begg’s *p* = 0.72; Egger’s *p* = 0.90), red meat (Begg’s *p* = 0.90; Egger’s *p* = 0.09), processed meat (Begg’s *p* > 0.99; Egger’s *p* = 0.70), and white meat (Begg’s *p* = 0.22; Egger’s *p* = 0.13) intake.

## 4. Discussion

In this meta-analysis, a high intake of total meat, red meat and processed meat was associated with increased risk of metabolic syndrome. People in the highest category of total meat, red meat and processed meat intake had an increased risk of metabolic syndrome of 14%, 33%, and 35%, respectively, compared with those in the lowest intake category. On the other hand, white meat intake was inversely associated with metabolic syndrome risk. People who have a high consumption of white meat had a 14% reduced risk of metabolic syndrome compared to those who have a low consumption of white meat. All of these associations did not differ significantly by study design and adjustment factors. 

The evidence of heterogeneity between studies was found in the meta-analysis of red meat intake and risk of metabolic syndrome. The possible reason for the observed heterogeneity was found in the subgroup analysis by geographic region. The results from the Asian population showed an inverse association between red meat intake and metabolic syndrome unlike the results from other populations. The observed heterogeneity in the analysis of red meat consumption disappeared after excluding studies targeting the Asian population (*I*^2^ = 17.0%, *p* = 0.31) [33,35]. Moreover, the relationship between red meat intake and the risk of metabolic syndrome became stronger when these studies were excluded (RR = 1.71, 95% CI: 1.37, 2.12). This result is consistent with the result from a recent meta-analysis of CVD mortality and red meat intake, which showed a stronger positive association after excluding Asian studies [8]. Relatively low red meat consumption in Asian population compared to Western population might explain the different results [36].

Several meta-analyses reported that a high intake of red and processed meat would raise the risk of diabetes [6,37], CVD [7,8], cancers [9,38,39], and mortality [8,40]. One portion per day increase in red and processed meat intake had a 14 and 32% increased risk, respectively, of type 2 diabetes [6]. In the case of mortality, high processed meat consumption was associated with a high risk of mortality from all-causes and CVD of 22% and 18%, respectively [8]. Also, people in the highest category of red meat intake had 29% and 16% increased risk of death from all-causes and CVD, respectively [8,40]. These findings are in agreement with our results in that a high intake of both red and processed meat might have a harmful effect on health. There were a relatively small number of studies that reported the relationship between white meat consumption and risk of disease. One meta-analysis including 4 studies suggested that a high intake of white meat was related to a reduction of total mortality of 5% in women, and they found a non-significant inverse association in relation to mortality from CVD and ischemic heart disease [8]. In addition, a pooled analysis of 8 Asian prospective cohort studies reported a lower risk of total mortality in people with high poultry intake [36]. More studies examining the association between white meat and the risk of diseases including metabolic syndrome are required to identify effects of white meat consumption on the incidence and development of disease.

In our nationally representative study including 8387 Korean adults aged 19–64 years, the intake of red and processed meat was not significantly associated with the prevalence of metabolic syndrome. These non-significant associations are similar to previous results from Asian studies. A cross-sectional study on the Thai population reported a non-significant association between meat intake and the prevalence of metabolic syndrome in men (OR = 1.01, 95% CI: 0.82, 1.23) and women (OR = 0.94, 95% CI: 0.72, 1.21) in the analysis of the highest vs. lowest quartile of meat intake [15]. A pooled analysis of 8 Asian prospective cohort studies also found a non-significant inverse association or significant inverse association between total or red meat consumption and death from CVD, cancer, or all-causes in male and female [36]. These results are in contrast with our meta-analysis results, which showed positive associations between total and red meat consumption and the risk of metabolic syndrome. This difference in the effect of meat consumption might be attributable to the lower intakes of red meat in Asian countries compared to Western countries [36]. There were a relatively small number of studies which examined the relationship between meat intake and metabolic syndrome in the Asian population compared to the Western population, and thus more well-designed studies targeting large populations are warranted to identify the effect of meat intake on metabolic syndrome in Asian countries. For processed meat consumption, we found that people in the highest quintile had a 32% higher prevalence of hyperglycemia compared to those in the lowest quintile. These results correspond to former studies, which reported an increased risk of diabetes by high processed meat intake [41,42]. We did not observe a significant association between white meat intake and the prevalence of metabolic syndrome, but inverse associations were observed in relation to the prevalence of hypertriglyceridemia and elevated blood pressure. Korean adults in the highest quintile of white meat intake had a lower prevalence of hypertriglyceridemia and elevated blood pressure, i.e., 24% and 32%, respectively, compared to those in the lowest quintile of white meat intake. There is a possibility that high white meat consumption was associated with low red meat intake, and this fact could have affected the results. However, we observed that people with high white meat intake also consumed more red meat, and the inverse association between white meat intake and hypertriglyceridemia and elevated blood pressure remained after further adjustment for dietary intakes including red meat.

Some potential mechanisms might explain a harmful effect of red and processed meat intake on metabolic syndrome. Red meat contains large amounts of total fat, saturated fat and haem-iron. Total and saturated fat might increase metabolic syndrome risk by obesity, hyperinsulinaemia and hyperglycemia [43,44]. Iron is a strong pro-oxidant, and thus can promote oxidative stress, which can damage tissues such as pancreatic beta cells. Furthermore, a high iron level may inhibit glucose metabolism and decrease synthesis and secretion of pancreatic insulin [37,45]. Nitrate used as a preservative in processed meat can be converted into nitrosamines. Nitrosamines have been observed to be poisonous to pancreatic cells and result in insulin resistance [8,46]. High levels of inflammatory mediators including C-reactive protein in people with high red and processed meat consumption also might be another reason for the increased risk of metabolic syndrome [18,47]. Unlike red meat, white meat contains a high proportion of polyunsaturated fatty acids and a low proportion of saturated fatty acids [10]. These differences in fat content might be the reason for the opposite effect of red and white meat on the risk of metabolic syndrome. In addition, the positive association between total meat consumption and metabolic syndrome, which was observed in the current meta-analysis, might be attributable to the high proportion of red meat in meat intake.

To the best of our knowledge, this is the first comprehensive meta-analysis to examine the relationship between total, red, processed and white meat consumption and the risk of metabolic syndrome. We conducted the analyses by types of meat. Most of the studies that were included in the meta-analysis were controlled for confounding factors including age, alcohol consumption, smoking, or physical activity. In addition, the relationships of total, white, red, and processed meat consumption and metabolic syndrome risk did not substantially differ by study design and adjustment factors, and we found no evidence of publication bias in all analyses. 

Despite these strengths, there are several limitations which should be considered. First, most of the studies that were included in our meta-analysis evaluated meat intake using FFQ, and thus, our results are susceptible to some degree of misclassification. However, the misclassification in the evaluation of exposure would more likely be non-differential, and the results would have biased toward the null. Second, a meta-analysis of observational studies cannot address problems of confounders that could be inherent in the original studies. Although most of the studies included in the meta-analysis controlled for other known risk factors including age, alcohol consumption, smoking or physical activity, residual confounding cannot be totally ruled out. People consuming more meat tended to have a low fruit and vegetable consumption. Some of the studies included in the meta-analysis controlled other food intake, but other studies did not. More well-designed studies that include various food consumption as an adjustment variable are required to confirm the association between meat consumption and the risk of metabolic syndrome. Third, because our study in Korean adults had a cross-sectional design, which assessed information of exposure and outcome simultaneously, the direction of causality cannot be inferred. However, we tried to eliminate a possible reverse causation bias by excluding both participants with a self-reported diagnosis of hypertension, diabetes, dyslipidemia, stroke, myocardial infarction, or cancer and those who were taking medications to control diabetes, hypertension, or dyslipidemia. Lastly, the results of the Korean adults were significant only in relation to the components of the metabolic syndrome such as hypertriglyceridemia, elevated blood pressure, and hyperglycemia.

## 5. Conclusions

In conclusion, we observed that total, red, and processed meat consumption was associated with a high risk of metabolic syndrome, while white meat consumption was inversely associated with the risk of metabolic syndrome. White meat intake was inversely associated with the prevalence of hypertriglyceridemia and elevated blood pressure, and high processed meat intake was associated with the raised prevalence of hyperglycemia in Korean adults. Our findings suggest that the effect on health is different by the types of meat and also support current common dietary guidelines to decrease the intake of red and processed meat and increase the consumption of white meat. The results of this study need to be confirmed in well-designed prospective cohort studies and randomized controlled trials on a large population.

## Figures and Tables

**Table 1:** Characteristics of epidemiological studies included in the meta-analysis of meat consumption and metabolic syndrome.

First Author (year)	Country	Study Design	Age (years)	Subjects	Criteria for Metabolic Syndrome	MEAT Consumption	Relative Risk (95% CI)	Adjustment for Covariates
Damiao (2006) [22]	Brazil	Cohort	30–64	57/151	NCEP ATP III	Red meat (g/day) 144.2 vs. 19.5	3.18 (0.87, 11.5)	Age, sex, physical activity, smoking, education level, alcohol, total energy intake, total fat intake, fried foods
						White meat (g/day) 28.7 vs. 4.6	1.36 (0.38, 4.78)	
Lutsey (2008) [21]	USA	Cohort	45–64	3782/9514	American Heart Association guidelines	Total meat (servings/day) 1.94 vs. 0.25	1.26 (1.11, 1.43)	Age, sex, race, education, center, total calories, smoking status, pack-years, physical activity, intakes of meat, dairy, fruits and vegetables, whole grains, and refined grains
Baik (2013) [35]	Korea	Cohort	40–69	1325/5251	The definition given by Alberti et al. [3]	Red meat (servings/day) 1.0 vs. 0	1.01 (0.79, 1.29)	Age, sex, income, occupation, education, smoking status, alcohol intake, quartiles of MET-hours/day, study sites, FTO genotypes, quartiles of energy intake, quintiles of healthy dietary pattern, unhealthy dietary pattern, refined grains and starches, mixed grain rice and cereal, fish and other seafood, eggs, legumes, nuts, vegetables and seaweed, fruits, dairy products, sweetened carbonated beverage, green tea and coffee. Types of meats were mutually adjusted for each other.
						White meat (servings/day) 0.4 vs. 0	0.88 (0.71, 1.09)	
Becerra-Tomas (2016) [19]	Spain	Cohort	55–80 (male) 60–80 (female)	930/1868	The definition given by Alberti et al. [3]	Tertiles (g/day) Total meat 158.9 vs. 87.0	1.23 (1.03, 1.45)	Age, sex, intervention group, leisure time physical activity, BMI, current smoker, former smoker, average consumption quintiles of vegetables, fruit, legumes, cereals, fish, dairy products, alcohol, biscuits, olive oil and nuts, the prevalence of metabolic syndrome components at baseline
						Red meat 96.4 vs. 38.4	1.46 (1.22, 1.74)	
						Processed meat 35.3 vs. 12.3	1.37 (1.15, 1.62)	
						White meat 79.4 vs. 28.9	0.83 (0.70, 0.99)	
Mennen (2000) [13]	France	Cross-sectional	30–64	1601/4976	The presence of at least two of the following factors in the upper (or lower in the case of HDL cholesterol) sex-specific quartile: fasting glucose, serum triglycerides, HDL cholesterol and DBP.	Total meat (portion/day) >2 vs. <1	Male 1.39 (0.92, 2.28)	Age, waist- hip ratio, energy intake
							Female 1.05 (0.67, 1.65)	
Yen (2006) [32]	China	Cross-sectional	30–79	3957/19,839	NCEP ATP III	Total meat (times/day) ≥3 vs. never or seldom	1.13 (1.08, 1.18)	Age, betel-quid chewing habit, education level, physical activity, occupation, smoking habit, alcohol habit, dietary intake, family history of diabetes, hypertension, cerebrovascular and CVD in second degree relatives
Ruidavets (2007) [24]	France	Cross-sectional	45–64 (male)	214/912	NCEP ATP III	Quintiles (g/day) Total meat 29.7 vs. 20.0	2.29 (1.30, 4.02)	Age, physical activity, centre, level of education, alcohol intake, smoking habits, drugs for dyslipidaemia and hypertension, energy intake (without alcohol), diet quality index, dieting
Azadbakht (2009) [18]	Iran	Cross-sectional	18–74 (female)	145/482	NCEP ATP III	Red meat (g/day) ≥63.7 vs. <27.3	1.99 (1.09, 3.89)	Age, physical activity, total energy intake, current estrogen use, menopausal status, family history of diabetes or stroke, intakes of dietary fiber and cholesterol, percent of energy from fat, fruit, and vegetables, white meats and fish, dairy, partially hydrogenated and nonhydrogenated vegetable oils, and whole- and refined-grains, BMI
Kouki (2011) [17]	Finland	Cross-sectional	57–78	351/1334	NCEP ATP III	Processed meat (g/day) Male >42.5 vs. 0	1.38 (0.85, 2.22)	Age, smoking, alcohol consumption, education and VO2 max
						Female >23.0 vs. 0	1.72 (1.08, 2.74)	
Babio (2012) [16]	Spain	Cross-sectional	55–80 (male), 60–80 (female)	447/717	NCEP ATP III	Quartiles (g/day) Red meat 150.6 vs. 35.9	2.3 (1.4, 3.9)	Age, sex, smoking, BMI, physical activity, total energy intake, dietary baseline variables (alcohol, dietary fibre, magnesium and potassium)
Strand (2015) [23]	China	Cross-sectional	44–52	368/793	NCEP ATP III	Total meat Often vs. rarely	0.9 (0.6, 1.4)	Unadjusted
Aekplakorn (2015) [15]	Thailand	Cross-sectional	30–59	1268/5872	The definition given by Alberti et al. [3]	Total meat Q4 vs. Q1	Male 1.01 (0.82, 1.23)	Age, alcohol drinking, family history of diabetes and smoking, leisure time physical activity, BMI
							Female 0.94 (0.72, 1.21)	
Cocate (2015) [14]	Brazil	Cross-sectional	50.5 (male)	94/296	The definition given by Alberti et al. [3]	Tertiles (g/day) Red meat ≥81.5 vs. <56.0	1.90 (1.06, 3.44)	Age, habitual physical activity, smoking habit, excessive alcohol intake, daily caloric intake
						White meat ≥39.4 vs. <24.0	1.12 (0.64, 1.97)	
Kim (2017) [33]	Korea	Cross-sectional	30–64	3143/11,029	NCEP ATP III	Red meat T3 vs. T1	0.89 (0.79, 1.00)	Age, sex, total energy intake, diet modification(receipt of dietary advice), education level
KNHANES	Korea	Cross-sectional	19–64	1325/8387	NCEP ATP III	Quintiles (servings/week) Total meat 16.2 vs. 1.4	0.85 (0.59, 1.24)	Age, sex, household income, education, smoking, alcohol, total energy intake, survey year, physical activity, BMI, intakes of coffee, green tea, soda, vegetables, legumes, whole grains, fish, nuts, dairy.
						Red meat 9.6 vs. 0.8	0.84 (0.59, 1.21)	
						Processed meat 3.1 vs. 0.0	1.18 (0.9, 1.56)	
						White meat 3.8 vs. 0.0	0.80 (0.58, 1.09)	
Chiu (2007) [34]	Taiwan	Case-control	≥20	572 cases/4690 controls	NCEP ATPIII	Total meat Usually vs. infrequent	1.08 (0.89, 1.30)	Age, gender, place of residence of case and control proband

Abbreviations: NCEP ATP III, National Cholesterol Education Program Adult Treatment Panel III; KNHANES, Korea National Health and Nutrition Examination Survey; DBP, diastolic blood pressure.

**Table 2:** Summary of pooled relative risks for meat consumption and risk of metabolic syndrome for highest vs. lowest meat consumption.

Factor	No. of Studies	Relative Risk	95% CIs	*p* for Difference
***Total meat***				
All studies	9	1.14	1.05, 1.23	
**Stratified by study design**				
Cohort study	2	1.25	1.13, 1.38	
Cross-sectional study	6	1.09	0.96, 1.24	0.17 ^a^
Case-control study	1	1.08	0.89, 1.31	0.33 ^a^
**Stratified by geographical region**				
Asia	5	1.11	1.06, 1.16	
Europe	3	1.36	1.04, 1.78	0.10 ^b^
North America	1	1.26	1.11, 1.43	0.14 ^b^
**Adjusted for BMI**				
Yes	2	1.07	0.91, 1.26	0.52
No	7	1.16	1.05, 1.28	
**Adjusted for smoking, alcohol and physical activity**				
Yes	5	1.11	0.97, 1.26	0.41
No	4	1.20	1.09, 1.32	
**Meat intake assessment**				
Grams ^c^	2	1.58	0.87, 2.87	0.19
Servings ^d^	7	1.12	1.05, 1.19	
***Red meat***				
All studies	8	1.33	1.01, 1.74	
**Stratified by study design**				
Cohort study	3	1.31	0.91, 1.89	0.97
Cross-sectional study	5	1.36	0.90, 2.07	
**Stratified by geographical region**				
Asia	3	0.91	0.82, 1.00	
Europe	2	1.72	1.12, 2.63	0.01 ^e^
South America	2	2.08	1.22, 3.55	0.04 ^e^
Middle East	1	1.99	1.05, 3.76	0.08 ^e^
**Adjusted for BMI**				
Yes	4	1.47	0.99, 2.18	0.60
No	4	1.13	0.83, 1.55	
**Adjusted for smoking, alcohol and physical activity**				
Yes	5	1.54	1.06, 2.24	0.33
No	3	1.04	0.79, 1.38	
**Meat intake assessment**				
Grams ^c^	5	1.71	1.37, 2.12	0.002
Servings ^d^	3	0.91	0.82, 1.00	
***White meat***				
All studies	5	0.86	0.76, 0.97	
**Stratified by study design**				
Cohort study	3	0.85	0.75, 0.98	0.93
Cross-sectional study	2	0.87	0.65, 1.16	
**Stratified by geographical region**				
South America	2	1.16	0.69, 1.93	
Europe	1	0.83	0.70, 0.99	0.35 ^f^
Asia	2	0.85	0.72, 1.02	0.39 ^f^
**Adjusted for BMI**				
Yes	2	0.82	0.71, 0.96	0.46
No	3	0.92	0.75, 1.12	
**Adjusted for smoking, alcohol and physical activity**				
Yes	4	0.85	0.73, 0.98	0.78
No	1	0.88	0.71, 1.09	
**Meat intake assessmen**				
Grams ^c^	3	0.86	0.73, 1.01	0.97
Servings ^d^	2	0.85	0.72, 1.02	

^a^ *p* value for difference in RRs of total meat consumption for cross-sectional study vs. cohort study (*p* = 0.17) and case-control study vs. cohort study (*p* = 0.33). ^b^ *p* value for difference in RRs of total meat consumption for Europe vs. Asia (*p* = 0.10) and North America vs. Asia (*p* = 0.14). ^c^ Studies assessed meat intakes by grams. ^d^ Studies assessed meat intake by servings or frequencies ^e^ *p* value for difference in RRs of red meat consumption for Europe vs. Asia (*p* = 0.01), South America vs. Asia (*p* = 0.04), and Middle East vs. Asia (*p* = 0.08). ^f^ *p* value for difference in RRs of white meat consumption for Europe vs. South America (*p* = 0.35) and Asia vs. South America (*p* = 0.39).

## Supplementary Materials

The following are available online at http://www.mdpi.com/2072-6643/10/4/390/s1, Table S1, Demographic and dietary intake profiles according to meat consumption in adults (KNHANES)^a^. Table S2, Multivariate adjusted odds ratio and 95% CI values for metabolic syndrome components according to white meat consumption. Table S3, Multivariate adjusted odds ratio and 95% CI values for metabolic syndrome components according to red meat consumption. Table S4, Multivariate adjusted odds ratio and 95% CI values for metabolic syndrome components according to processed meat consumption.
