# Supplementary material for: Meat Consumption and Risk of Metabolic Syndrome: Results from the Korean Population and a Meta-Analysis of Observational Studies
Source: Nutrients. 2018 Mar 22;10(4):390. doi: 10.3390/nu10040390 (PMC5946175; doi:10.3390/nu10040390)
Supplement: Supplementary file 1 [file nutrients-10-00390-s001.zip › Supplement Data(KNHANES METHODS).docx]

**Description of the Korea National Health and Nutrition Examination Survey (KNHANES) study population and methods**

*Study participants*

The present study examines data from the KNHANES 2012-2015, which was conducted by Korea Centers for Disease Control and Prevention [1]. The KNHANES is a cross-sectional, nationally representative sample of the South Korean population. The KNHANES population was recruited using a clustered, multistage, stratified, and probability sampling design based on age-groups, gender and geographical region to represent the non-institutionalized civilian South Korean population. Through 3 surveys including a health examination, a health interview, and a nutrition survey, the KNHANES gathered information on health condition, attitudes, and behaviors related with health, diet, and nutrition annually. The data-collecting and physical examination method of KNHANES have been described in detail previously [1]. A total of 31,006 participants from 2012 (n= 8,058), 2013 (n=8,018), 2014 (n=7,550), and 2015 (n=7,380) were collected. Among them, those with complete data for the health examination, health interview, and nutrition survey were included. Candidates were sequentially excluded if they were not eligible for our study: 11,459 participants who were aged <19 or ≥65 years; 3,509 participants who had a self-reported diagnosis of diabetes, hypertension, hyperlipidemia, stroke, myocardial infarction, or cancer or were taking medications to control diabetes, hypertension, or hyperlipidemia; 96 participants who were pregnant; 678 participants who had missing information on fasting serum glucose, HDL-cholesterol, triglyceride, and systolic and diastolic blood pressure; 97 participants who had no fasting (<8 hours) status at blood test; 859 participants who had extreme energy intake (≤500 or >6000 kcal/day); participants with missing data on alcohol consumption (n=996) and moderate or vigorous physical activity (n= 182). Finally, 8,387 participants (2,275 in 2012, 2,233 in 2013, 1,962 in 2014, and 1,917 in 2015) were included in the analysis of metabolic syndrome. Human subject procedures for KNHANES were approved by the Institutional Review Board (IRB) of Korea Centers for Disease Control and Prevention (2012-01EXP-01-2C, 2013-07CON-03-4C, 2013-12EXP-03-5C, 2015-01-02-6C), and informed consent was obtained from each subject in the survey.

*Dietary intake assessment*

Dietary consumption was assessed using the 112-item dish-based semi-quantitative food frequency questionnaires (FFQ) of KNHANES. The validity and reproducibility of the semi-quantitative FFQ were evaluated by using 3-day diet records, and the details have been described previously [2]. The FFQ contained information on frequency of consumption of foods or dishes over the last year, along with serving sizes. The frequency category consisted of multiple choices ranging from “almost never” to ‘3 times per day.” Meat consumption was estimated through multiplying the frequency of meat intake by the serving size of meat intake. Trained interviewers helped participants to choose the serving size. The serving size options were composed of three categories (a half of, equal to, and 1.5 or 2 times of a standard serving size). The meat consumption was assessed from 16 food items (stock soup of bone and stew meat/beef-bone soup/bone soup, pork back-bone stew, beef stew/hot spicy meat stew/radish soup, grilled pork belly, boiled pork, stir-fried spicy pork/pork bulgogi/grilled spareribs/steamed pork ribs, sweet and sour pork/pork cutlet, roast beef, beef bulgogi, sausage stew, ham, sundae, chicken soup with ginseng, chopped roast chicken/boiled chicken, fried chicken, grilled duck) in the FFQ. The red meat group included stock soup of bone and stew meat/beef-bone soup/bone soup, pork back-bone stew, beef stew/hot spicy meat stew/radish soup, grilled pork belly, boiled pork, stir-fried spicy pork/pork bulgogi/grilled spareribs/steamed pork ribs, sweet and sour pork/pork cutlet, roast beef, beef bulgogi, sausage stew, ham, and sundae. The processed meat group included sausage stew, ham, and sundae. The white meat group included chicken soup with ginseng, chopped roast chicken/boiled chicken, fried chicken, and grilled duck.

*Assessment of metabolic syndrome*

The definition of metabolic syndrome was based on the harmonizing criteria by Aberti *et al* [3]. The metabolic syndrome was diagnosed when three or more of following components were present: 1) abdominal obesity (waist circumference ≥90 cm for men and ≥80 cm for women); 2) hypertriglyceridemia (triglycerides ≥150 mg/dL); 3) hyperglycemia (fasting serum glucose ≥100 mg/dL); 4) low HDL cholesterol (HDL cholesterol <40 mg/dL for men and <50 mg/dL for women); and 5) elevated blood pressure (systolic blood pressure ≥130 mmHg or diastolic blood pressure ≥85 mmHg).

*Confounding variables*

A self-administered questionnaire was used to collect information on the sociodemographic factors including age, sex, and socioeconomic status. Household income was categorized as quartile, and education level was classified as “college or higher,” “middle or high school,” and “elementary school or lower.” The various health behaviors including smoking status, physical activity, and alcohol consumption were also self-reported. Smoking status was categorized into non-smoker, ex-smoker, and current smoker. Physical activity was assessed by the practice of aerobic activities. Those who with ≥150 minutes/week of moderate activity, ≥75 minutes/week of vigorous activity, or ≥150 minutes/week of a combination of moderate and vigorous activity (1 minute of vigorous activity was calculated to 2 minutes of moderate activity) were considered physically active. The weekly servings of alcoholic beverages were estimated through multiplying the frequency of alcohol drinking by the amount of alcoholic beverage. Alcohol consumption was categorized as < 1 serving per day, 1-2 servings per day, and ≥3 servings per day. Besides above variables, there were other covariates such as age (year, continuous), sex, BMI (continuous), survey year (2012, 2013, 2014, 2015) and total energy intake (kcal, continuous).

*Statistical analysis*

The age-standardized mean and prevalence of demographic factors and dietary intakes were compared according to meat consumption with use of the PROC SURVEYREG and PROC SURVEYLOGISTIC (SAS Institute) analysis taking into account the clustering sampling design of the survey. Food consumption was estimated by multiplying the frequency of food intake by the amount of servings consumed for each food item. The median values of each category of food consumption frequency were used to calculate the frequency of food intake (‘None’ = 0, ‘1 time/month’ = 1/4.3, ‘2-3 times/month’= 2.5/4.3, ‘1 time/week’ = 1, ‘2-4 times/week’ = 3, ‘5-6 times/week’ = 5.5, ‘7 times/week’ = 7, ‘14 times/week’ = 14, ‘21 times/week’ = 21). The amount of servings consumed was converted to the ratio in proportion to the standard serving size. The participants were classified into quintiles based on meat intake to evaluate the association between meat consumption and metabolic syndrome. In model 1, we adjusted for the following confounders: age (continuous), sex, BMI (continuous), education level (elementary school or lower; middle or high school; college or higher), smoking (non-smoker; ex-smoker; current smoker), alcohol consumption (< 1 serving/day; 1-2 servings/day; ≥3 servings/day), physical activity (practice of aerobic activities), survey year (2012; 2013; 2014; 2015), household income (quartile) and total energy intake (kcal, continuous). We further mutually adjusted for consumption of vegetables, fruit, fish, dairy, legumes, nuts, whole grains, coffee, soda, and green tea consumption in the model 2 (continuous, servings/week). Linear trends across meat consumption categories were estimated by assigning the median value within each exposure category with use of the PROC SURVEYLOGISTIC analysis. All statistical analyses were performed with SAS software (version 9.4; SAS Institute, Cary, NC). A *P* value < 0.05 was considered statistically significant.

**Supplemental references**

1. Kweon, S.; Kim, Y.; Jang, M.J.; Kim, Y.; Kim, K.; Choi, S.; Chun, C.; Khang, Y.H.; Oh, K. Data resource profile: The korea national health and nutrition examination survey (knhanes). Int J Epidemiol **2014**, 43, 69-77.
2. Kim, D.W.; Song, S.; Lee, J.E.; Oh, K.; Shim, J.; Kweon, S.; Paik, H.Y.; Joung, H. Reproducibility and validity of an ffq developed for the korea national health and nutrition examination survey (knhanes). Public health nutrition **2015**, 18, 1369-1377.
3. Alberti, K.G.; Eckel, R.H.; Grundy, S.M.; Zimmet, P.Z.; Cleeman, J.I.; Donato, K.A.; Fruchart, J.C.; James, W.P.; Loria, C.M.; Smith, S.C., Jr., et al. Harmonizing the metabolic syndrome: A joint interim statement of the international diabetes federation task force on epidemiology and prevention; national heart, lung, and blood institute; american heart association; world heart federation; international atherosclerosis society; and international association for the study of obesity. Circulation **2009**, 120, 1640-1645.
