# Supplementary material for: Meat Consumption and Risk of Metabolic Syndrome: Results from the Korean Population and a Meta-Analysis of Observational Studies
Source: Nutrients. 2018 Mar 22;10(4):390. doi: 10.3390/nu10040390 (PMC5946175; doi:10.3390/nu10040390)
Supplement: Supplementary file 1 [file nutrients-10-00390-s001.zip › Supplementary Table 1.docx]

**Supplementary Table 1**. Demographic and dietary intake profiles according to meat consumption in adults (KNHANES)^a^

|  | **Red meat** | | **Processed meat** | | **White meat** | |
| --- | --- | --- | --- | --- | --- | --- |
|  | **Quintile 1** | **Quintile 5** | **Quintile 1** | **Quintile 5** | **Quintile 1** | **Quintile 5** |
| **Men, n** | 678 | 672 | 949 | 678 | 675 | 672 |
| Age,^b^ y | 45.1±0.5 | 32.9±0.4 | 45.1±0.4 | 32.5±0.4 | 44.9±0.5 | 33.3±0.4 |
| Energy intake,^c^ kcal/d | 1838.9±27.8 | 3350.7±36.0 | 2168.0±30.6 | 2998.7±37.3 | 1996.2±30.9 | 3138.6±35.4 |
| BMI,^c^ kg/m^2^ | 24.0±0.1 | 24.3±0.2 | 24.1±0.1 | 24.3±0.2 | 24.0±0.2 | 24.2±0.1 |
| High physical activity,^d, f^ % | 56.4 | 55.1 | 58.0 | 55.0 | 47.8 | 47.5 |
| Alcohol, ≥1 drink/d,^f^ % | 30.1 | 46.5 | 37.7 | 43.4 | 33.9 | 48.7 |
| High education,^e, f %^ | 34.4 | 47.7 | 34.9 | 48.4 | 36.8 | 44.9 |
| Currently smoking,^f^% | 38.2 | 51.6 | 42.8 | 47.8 | 47.6 | 43 |
| Highest income quartile, ^f^ % | 26.4 | 39.7 | 32.3 | 37.0 | 30.9 | 43.1 |
| Dietary intake |  |  |  |  |  |  |
| Vegetables,^c^ servings/week | 10.7±0.5 | 25.7±0.6 | 15.0±0.5 | 21.9±0.6 | 14.5±1.2 | 24.2±1.2 |
| Fruit,^c^ servings/week | 6.0±0.3 | 10.6±0.4 | 7.2±0.2 | 9.4±0.3 | 6.6±0.7 | 10.6±0.6 |
| Legumes,^c^ servings/week | 3.0±0.2 | 6.7±0.2 | 4.2±0.2 | 5.9±0.2 | 3.8±0.2 | 5.9±0.2 |
| Whole grains,^c^ servings/week | 8.2±0.4 | 7.1±0.3 | 8.0±0.3 | 7.1±0.3 | 8.8±0.7 | 8.4±0.6 |
| White meat,^c^ servings/week | 1.0±0.1 | 3.8±0.1 | 1.5±0.1 | 3.1±0.1 | 3.2±0.3 | 9.2±0.5 |
| Fish,^c^ servings/week | 2.6±0.2 | 5.6±0.2 | 3.7±0.2 | 4.9±0.2 | 3.5±0.6 | 6.2±0.5 |
| Dairy,^c^ servings/week | 4.3±0.3 | 6.0±0.3 | 4.9±0.3 | 5.7±0.3 | 4.9±0.6 | 6.0±0.5 |
| Nuts,^c^ servings/week | 0.4±0.1 | 0.9±0.1 | 0.5±0.1 | 0.8±0.1 | 0.4±0.1 | 1.0±0.2 |
| Coffee,^c^ times/week | 10.7±0.6 | 16.4±0.7 | 12.3±0.6 | 14.8±0.6 | 13.8±1.3 | 15.7±1.3 |
| Soda,^c^ cups/week | 1.3±0.1 | 2.4±0.2 | 1.5±0.1 | 2.1±0.1 | 1.3±0.3 | 2.4±0.3 |
| Green tea,^c^ cups/week | 1.2±0.2 | 1.3±0.1 | 1.3±0.2 | 1.3±0.1 | 1.7±0.5 | 1.3±0.2 |
| **Women, n** | 981 | 1006 | 1673 | 1005 | 939 | 1003 |
| Age,^b^ y | 45.1±0.4 | 33.4±0.4 | 44.6±0.4 | 32.9±0.3 | 45.3±0.4 | 33.4±0.4 |
| Energy intake,^c^ kcal/d | 1438.4±19.7 | 2576.0±27.8 | 1676.3±18.3 | 2360.4±30.7 | 1588.5±24.4 | 2444.8±28.8 |
| BMI,^c^ kg/m^2^ | 22.7±0.1 | 22.7±0.1 | 22.4±0.1 | 22.8±0.1 | 22.4±0.2 | 22.7±0.1 |
| High physical activity,^d, f^ % | 38.5 | 44.1 | 42.9 | 42.6 | 38 | 44.8 |
| Alcohol, ≥1 drink/d, ^f^ % | 7.9 | 18 | 10.4 | 15.4 | 8 | 16.9 |
| High education,^e, f^ % | 34.6 | 46.1 | 34.9 | 45.2 | 36.6 | 43.7 |
| Currently smoking,^f^ % | 6.8 | 9.9 | 7.3 | 7.7 | 8.3 | 6.7 |
| Highest income quartile, ^f^ % | 30.4 | 37.5 | 35.7 | 34.7 | 30.2 | 38.7 |
| Dietary intake |  |  |  |  |  |  |
| Vegetables,^c^ servings/week | 12.6±0.5 | 23.1±0.5 | 16.0±0.4 | 21.0±0.5 | 14.3±0.5 | 21.8±0.4 |
| Fruit,^c^ servings/week | 8.5±0.3 | 12.5±0.3 | 10.1±0.3 | 11.0±0.3 | 8.5±0.3 | 12.4±0.3 |
| Legumes,^c^ servings/week | 3.3±0.1 | 5.9±0.2 | 4.0±0.1 | 5.4±0.2 | 3.9±0.2 | 5.5±0.1 |
| Whole grains,^c^ servings/week | 7.7±0.2 | 7.2±0.2 | 7.8±0.2 | 7.3±0.2 | 7.8±0.3 | 7.4±0.2 |
| White meat,^c^ servings/week | 0.9±0.1 | 3.0±0.1 | 1.3±0.1 | 2.6±0.1 | 2.4±0.1 | 6.6±0.2 |
| Fish,^c^ servings/week | 3.0±0.2 | 5.7±0.3 | 3.7±0.2 | 5.0±0.2 | 3.5±0.2 | 4.9±0.2 |
| Dairy,^c^ servings/week | 4.4±0.2 | 6.5±0.2 | 5.1±0.2 | 6.1±0.2 | 4.7±0.3 | 6.6±0.2 |
| Nuts,^c^ servings/week | 0.4±0.0 | 0.7±0.0 | 0.5±0.0 | 0.6±0.1 | 0.4±0.1 | 0.8±0.1 |
| Coffee,^c^ times/week | 8.4±0.4 | 11.2±0.4 | 8.5±0.3 | 11.1±0.4 | 9.1±0.4 | 10.0±0.3 |
| Soda,^c^ cups/week | 0.6±0.0 | 1.4±0.1 | 0.7±0.0 | 1.2±0.1 | 0.6±0.1 | 1.4±0.1 |
| Green tea,^c^ cups/week | 0.8±0.1 | 1.2±0.1 | 0.9±0.1 | 1.3±0.1 | 0.8±0.1 | 1.3±0.1 |

^a^ KNHANES, Korean National Health and Nutrition Examination Survey; BMI, body mass index.

^b^ Values are means ± SEMs

^c^ Values are age-standardized means±SEMs

^d^ High physical activity was defined as 150 minutes/week of moderate activity, ≥75 minutes/week of vigorous activity or ≥150 minutes/week of a combination of moderate and vigorous activity.

^e^ College education or higher.

^f^ Values are age-standardized prevalences.
