# Supplementary material for: Meat Consumption and Risk of Metabolic Syndrome: Results from the Korean Population and a Meta-Analysis of Observational Studies
Source: Nutrients. 2018 Mar 22;10(4):390. doi: 10.3390/nu10040390 (PMC5946175; doi:10.3390/nu10040390)
Supplement: Supplementary file 1 [file nutrients-10-00390-s001.zip › Supplementary Table 4.docx]

**Supplementary Table 4.** Multivariate adjusted odds ratio and 95% CI values for metabolic syndrome components according to processed meat consumption.

| **Processed meat consumption** | | | | | | |
| --- | --- | --- | --- | --- | --- | --- |
|  | **Quintile 1** | **Quintile 2** | **Quintile 3** | **Quintile 4** | **Quintile 5** | ***P*- trend** |
| **Metabolic syndrome** |  |  |  |  |  |  |
| All |  |  |  |  |  |  |
| No.of MetS/subjects | 514/2622 | 94/651 | 257/1726 | 224/1697 | 240/1691 |  |
| Median intake, servings/week | 0.0 | 0.2 | 0.7 | 1.4 | 3.1 |  |
| Model 1^a^ | 1.00 | 1.13 (0.83, 1.55) | 0.03 (0.82, 1.30) | 0.98 (0.76, 1.26) | 1.18 (0.90, 1.54) | 0.308 |
| Model 2^b^ | 1.00 | 1.14 (0.83, 1.56) | 1.03 (0.82, 1.30) | 0.99 (0.76, 1.28) | 1.18 (0.90, 1.56) | 0.307 |
| Men |  |  |  |  |  |  |
| No.of MetS/subjects | 220/949 | 80/445 | 116/544 | 137/743 | 126/678 |  |
| Median intake, servings/week | 0.0 | 0.3 | 0.7 | 1.6 | 3.6 | 0.937 |
| Model 1^a^ | 1.00 | 1.04 (0.71, 1.51) | 1.25 (0.89, 1.77) | 1.03 (0.72, 1.47) | 1.04 (0.71, 1.52) | 0.877 |
| Model 2^b^ | 1.00 | 1.05 (0.72, 1.52) | 1.23 (0.87, 1.75) | 1.02 (0.72, 1.45) | 1.03 (0.69, 1.52) |  |
| Women |  |  |  |  |  |  |
| No.of MetS/subjects | 294/1673 | 39/340 | 117/1015 | 93/995 | 107/1005 |  |
| Median intake, servings/week | 0.0 | 0.2 | 0.6 | 1.2 | 3.0 |  |
| Model 1^a^ | 1.00 | 1.03 (0.65, 1.63) | 0.84 (0.6, 1.18) | 1.06 (0.74, 1.52) | 1.38 (0.92, 2.06) | 0.073 |
| Model 2^b^ | 1.00 | 1.06 (0.67, 1.68) | 0.87 (0.62, 1.23) | 1.08 (0.75, 1.57) | 1.42 (0.94, 2.15) | 0.065 |
| **Abdominal obesity** |  |  |  |  |  |  |
| All |  |  |  |  |  |  |
| Model 1^a^ | 1.00 | 0.94 (0.65, 1.36) | 0.89 (0.67, 1.17) | 0.99 (0.74, 1.32) | 0.81 (0.59, 1.11) | 0.228 |
| Model 2^b^ | 1.00 | 0.94 (0.65, 1.37) | 0.89 (0.68, 1.18) | 0.99 (0.74, 1.32) | 0.82 (0.59, 1.14) | 0.285 |
| Men |  |  |  |  |  |  |
| Model 1^a^ | 1.00 | 1.05 (0.64, 1.71) | 0.91 (0.58, 1.41) | 0.83 (0.52, 1.33) | 0.61 (0.38, 0.99) | 0.021 |
| Model 2^b^ | 1.00 | 1.06 (0.65, 1.73) | 0.92 (0.59, 1.43) | 0.84 (0.52, 1.35) | 0.65 (0.40, 1.05) | 0.041 |
| Women |  |  |  |  |  |  |
| Model 1^a^ | 1.00 | 0.76 (0.45, 1.28) | 0.89 (0.64, 1.23) | 1.08 (0.76, 1.53) | 1.05 (0.72, 1.54) | 0.501 |
| Model 2^b^ | 1.00 | 0.75 (0.44, 1.26) | 0.89 (0.64, 1.23) | 1.09 (0.76, 1.55) | 1.05 (0.71, 1.55) | 0.535 |
| **Low HDL cholesterol** |  |  |  |  |  |  |
| All |  |  |  |  |  |  |
| Model 1^a^ | 1.00 | 1.20 (0.97, 1.48) | 1.00 (0.85, 1.18) | 1.05 (0.89, 1.26) | 1.17 (0.97, 1.41) | 0.140 |
| Model 2^b^ | 1.00 | 1.20 (0.97, 1.40) | 1.00 (0.85, 1.17) | 1.06 (0.89, 1.26) | 1.16 (0.96, 1.41) | 0.166 |
| Men |  |  |  |  |  |  |
| Model 1^a^ | 1.00 | 1.13 (0.82, 1.55) | 1.31 (0.97, 1.75) | 1.14 (0.86, 1.50) | 1.31 (0.97, 1.76) | 0.165 |
| Model 2^b^ | 1.00 | 1.13 (0.83, 1.54) | 1.30 (0.97, 1.75) | 1.14 (0.87, 1.51) | 1.31 (0.97, 1.78) | 0.157 |
| Women |  |  |  |  |  |  |
| Model 1^a^ | 1.00 | 1.07 (0.80, 1.42) | 0.92 (0.76, 1.12) | 0.95 (0.77, 1.18) | 1.08 (0.86, 1.37) | 0.403 |
| Model 2^b^ | 1.00 | 1.08 (0.81, 1.43) | 0.92 (0.75, 1.13) | 0.95 (0.77, 1.19) | 1.09 (0.86, 1.39) | 0.392 |
| **Hypertriglyceridemia** |  |  |  |  |  |  |
| All |  |  |  |  |  |  |
| Model 1^a^ | 1.00 | 1.04 (0.80, 1.33) | 0.95 (0.78, 1.15) | 0.95 (0.77, 1.16) | 1.05 (0.85, 1.30) | 0.573 |
| Model 2^b^ | 1.00 | 1.05 (0.81, 1.35) | 0.96 (0.79, 1.16) | 0.97 (0.79, 1.19) | 1.09 (0.87, 1.36) | 0.414 |
| Men |  |  |  |  |  |  |
| Model 1^a^ | 1.00 | 1.02 (0.76, 1.37) | 1.16 (0.89, 1.52) | 0.98 (0.74, 1.29) | 1.15 (0.87, 1.53) | 0.438 |
| Model 2^b^ | 1.00 | 1.04 (0.77, 1.39) | 1.17 (0.89, 1.54) | 0.99 (0.75, 1.31) | 1.18 (0.89, 1.58) | 0.350 |
| Women |  |  |  |  |  |  |
| Model 1^a^ | 1.00 | 1.00 (0.69, 1.44) | 0.80 (0.59, 1.09) | 0.84 (0.63, 1.12) | 0.91 (0.64, 1.28) | 0.699 |
| Model 2^b^ | 1.00 | 1.02 (0.70, 1.47) | 0.83 (0.61, 1.13) | 0.87 (0.65, 1.16) | 0.95 (0.66, 1.35) | 0.880 |
| **Hyperglycemia** |  |  |  |  |  |  |
| All |  |  |  |  |  |  |
| Model 1^a^ | 1.00 | 1.10 (0.85, 1.43) | 1.28 (1.05, 1.57) | 1.26 (1.02, 1.56) | 1.32 (1.04, 1.66) | 0.049 |
| Model 2^b^ | 1.00 | 1.10 (0.85, 1.43) | 1.28 (1.05, 1.55) | 1.24 (1.00, 1.54) | 1.27 (1.00, 1.60) | 0.113 |
| Men |  |  |  |  |  |  |
| Model 1^a^ | 1.00 | 1.03 (0.75, 1.43) | 1.14 (0.85, 1.54) | 1.15 (0.87, 1.54) | 1.20 (0.86, 1.68) | 0.302 |
| Model 2^b^ | 1.00 | 1.04 (0.76, 1.43) | 1.12 (0.83, 1.52) | 1.13 (0.85, 1.51) | 1.15 (0.83, 1.61) | 0.453 |
| Women |  |  |  |  |  |  |
| Model 1^a^ | 1.00 | 1.50 (1.05, 2.12) | 1.37 (1.05, 1.79) | 1.51 (1.10, 2.06) | 1.53 (1.10, 2.12) | 0.037 |
| Model 2^b^ | 1.00 | 1.52 (1.06, 2.17) | 1.41 (1.08, 1.84) | 1.50 (1.09, 2.08) | 1.50 (1.07, 2.11) | 0.068 |
| **Elevated blood pressure** |  |  |  |  |  |  |
| All |  |  |  |  |  |  |
| Model 1^a^ | 1.00 | 1.04 (0.78, 1.40) | 0.98 (0.79, 1.20) | 1.03 (0.84, 1.28) | 1.07 (0.84, 1.35) | 0.570 |
| Model 2^b^ | 1.00 | 1.06 (0.79, 1.42) | 0.99 (0.81, 1.22) | 1.07 (0.86, 1.32) | 1.11 (0.87, 1.42) | 0.371 |
| Men |  |  |  |  |  |  |
| Model 1^a^ | 1.00 | 1.02 (0.74, 1.42) | 0.93 (0.69, 1.24) | 0.91 (0.69, 1.20) | 1.08 (0.80, 1.47) | 0.578 |
| Model 2^b^ | 1.00 | 1.05 (0.76, 1.45) | 0.95 (0.71, 1.27) | 0.95 (0.72, 1.25) | 1.13 (0.83, 1.55) | 0.415 |
| Women |  |  |  |  |  |  |
| Model 1^a^ | 1.00 | 1.15 (0.79, 1.67) | 1.04 (0.79, 1.37) | 1.45 (1.05, 1.99) | 1.17 (0.80, 1.72) | 0.359 |
| Model 2^b^ | 1.00 | 1.15 (0.79, 1.68) | 1.06 (0.80, 1.41) | 1.46 (1.05, 2.03) | 1.21 (0.81, 1.80) | 0.310 |

^a^ Model 1 was adjusted for age, sex, household income, education, smoking, alcohol, total energy intake, survey year, physical activity, BMI.

^b^ Model 2 was adjusted for age, sex, household income, education, smoking, alcohol, total energy intake, survey year, physical activity, BMI, intakes of coffee, green tea, soda, vegetables, legumes, fruit, whole grains, fish, nuts, dairy and white meat.
